# Lifestyle Interventions to Promote Healthy Nutrition and Physical Activity in MiddleAge (40-60 Years) Adults: A Randomized Controlled Trial in the North of Iran

**Published:** 2019-01-09

**Authors:** Simin Mouodi, Seyed Reza Hosseini, Reza Ghadimi, Robert Graham Cumming, Ali Bijani, Marjan Mouodi, Yadollah Zahed Pasha

**Affiliations:** ^1^ Social Determinants of Health Research Center, Health Research Institute, Babol University of Medical Sciences, Babol, Iran; ^2^ Health Research Institute, Babol University of Medical Sciences, Babol, Iran; ^3^ School of Public Health, University of Sydney, Sydney, Australia; ^4^ Department of Internal Medicine, Babol University of Medical Sciences, Babol, Iran; ^5^ Non-communicable Pediatric Diseases Research Center, Babol University of Medical Sciences, Babol, Iran

**Keywords:** Lifestyle, Weight loss, Middle age, Diet

## Abstract

**Background:** This study was conducted to evaluate the effectiveness of short-term structured interventions on healthy lifestyle behaviors, dietary intake, anthropometric measures, blood pressure, fasting blood glucose, and serum lipid profile of middle-aged adults.

**Study design:** Randomized controlled trial study.

**Methods:** Overall, 300 individuals out of apparently healthy (non-patient) adults aged 40-60 yr living in Amirkola, Babol the north of Iran were enrolled in 2016-2017. The Persian translation of the Health Promoting Lifestyle Profile II (HPLP-II) and two days 24-h food recall questionnaires were used for data collection. Eligible participants were allocated randomly in three groups (high-intensive, low-intensive and the control). The follow-up examination has been conducted after 16 wk of intervention.

**Results:** The three study groups had no significant difference in age (*P*=0.888), sex (*P*=0.395), BMI (*P*=0.969), healthy lifestyle score (*P*=0.675) and total daily energy intake (*P*=0.612). After intervention, the mean scores of all the six subdomains of HPLP-II questionnaire had significant improvement (P*P*<0.001). Mean weight loss was 1.5, 1.0 and 0.3 kg, in high-intensive, low-intensive and control groups, respectively. BMI although reduced, was still in the overweight range in two sexes. Mean of neck, arm, waist and buttock size, diastolic blood pressure, serum triglyceride, total cholesterol, and HDL levels have been changed to a better condition in comparison with the baseline values (*P*<0.001). Dietary intake had good changes in total daily energy (*P*<0.001), daily intake of carbohydrate, protein, and fat (*P*<0.001), and proportion of energy from carbohydrate (*P*=0.007) and fat (*P*=0.022) after the intervention.

**Conclusion:** Our intervention program could have positive impact on healthy lifestyle behaviors, dietary intake and weight in addition to some other anthropometric variables and serum lipid profile of middleaged adults.

## Introduction

WHO encourages all government policy-makers to develop local, available and cost-effective approaches to prevent and control non-communicable diseases (NCDs), especially cardiovascular diseases, diabetes, cancer and chronic respiratory disorders, as the most important health threats in recent years ^1^. These major NCDs share four behavioral risk factors which represent the person´s lifestyle: tobacco use, unhealthy diet, physical inactivity and harmful use of alcohol^2, 3^.

Physical inactivity and unhealthy diets are major contributors to overweight and extra body weight increases the risk of serious health consequences such as cardiovascular diseases, diabetes as well as breast, colon and endometrial cancers; and some studies estimated the impact of excess body weight on cardiometabolic consequences^4^. At least 2.6 million people each year die as a result of being overweight or obese and 1.9 million deaths are attributable to physical inactivity^5^. Although unhealthy diet and sedentary life are difficult to manage because they have complex roots at the interface between individual choices and social and physical environments^6^, even obese individuals can lose approximately 6%-8% of initial weight with regular participation in a high-intensive lifestyle intervention consisting of diet, physical activity, and behavior therapy ^7^. Furthermore, weight loss could reduce the risk of hypertension 24%-40% in overweight and 40%-54% in obese people^8^.

Middle age (40-60 yr old) is an important period in the life cycle^9^. Time limitation and increased stress resulting from family and work responsibilities create important health challenges among middle-aged adults^10^. Maintaining good health lifestyle behaviors is particularly important for this age-group^11^.

In recent years, multiple studies have been conducted to evaluate the effectiveness of short and long-term lifestyle (especially diet and/or physical activity) modification programs in adult populations^12-15^. Each region has cultural demographics and individual characteristics which have an impact on the effectiveness of lifestyle modification programs^16, 17^.

We aimed to evaluate the effect of lifestyle modification interventions on nutritional behaviors, physical activity, anthropometric measurements, fasting blood sugar and serum lipid profile of adults aged 40-60 yr in Amirkola, Babol, the north of Iran.

## Methods

### Study design

This study is a part of a randomized population-based controlled trial conducted in the year 2016-2017 among the adults aged 40-60 yr living in Amirkola, Babol the north of Iran. Other data related to this study have been reported in previous manuscripts ^18, 19^.

### Participants

Inclusion criteria were age 40-60 yr; living in Amirkola; and desire to participate in the study; and exclusion criteria were unable to read and write; pregnancy or child breastfeeding; severe physical or mental disease leading to disability; probable migration from Amirkola in time duration of study (about 10 months); medical history of diabetes or hyperlipidemia which made the patient to have a specific diet or hypoglycemic and/or lipid-lowering agents.

To invite middle-age population, various methods were used such as: 1) informing local health care workers and family physicians; 2) public announcements by local health volunteers (non-employed women who have voluntary cooperation with health centers); and 3) distribution of public notices in households, public gathering places, mosques, banks, post offices, local markets, and stores. At first, the volunteers who referred to the research center have been checked for eligibility criteria, then, they were allocated randomly in three groups. At the same time, using the call number recorded in the health centers of the region, the researchers called the households which had at least one person in the age group of 40-60 yr and invited them to participate in the study.

### Interventions

Study population was allocated randomly in three groups: 1- high intensive intervention group 2- low intensive intervention group 3- control (education group). Intervention was continued for four months (from Jan to Apr 2017).

High intensive intervention consisted of weekly group nutrition and physical activity training classes, individual nutrition consultation, educational package and weekly aerobic exercises. Low intensive intervention consisted of weekly group nutrition training classes, individual nutrition consultation and educational package. Control group received only educational package. All sessions and individual consultations were free of charge and held by a general practitioner (Medical Doctor, Master of Public Health).

In weekly group sessions, participants were educated face-to-face in group discussions about the principles of correct nutrition behavior and physical activity. The content of these classes was derived from the standard educational packages of Ministry of Health and Medical Education of Iran and duration of each class was at least 60 min. An educated team managed these classes; this team included a medical doctor (main educator), an MSc in nutrition and a Ph.D. in clinical nutrition. The average number of participants invited to participate in each session was 25.

Weekly exercise classes consisted of free aerobic activities whose trainer was an educated person. We rented a sports hall in the center of research area to facilitate participation in these aerobic classes for the high intensive intervention group. The duration of each session was 60 min. Considering the cultural and religious values of this region, men and women had separate classes in different times; the sessions for women were held from 10:00 to 11:00 am on Sundays and Tuesdays and men´s classes were scheduled from 15:00 to 16:00 pm on Mondays and Wednesdays each week. Men had a male trainer and women had a female one.

An educational package (including a book and a DVD) provided specifically for this study, using the standard educational packages of Ministry of Health and Medical Education of Iran about the principles of nutrition and physical activity in middle-aged adults has been delivered to all of participants in the three groups^20^.

The Template for Intervention Description and Replication (TIDieR) checklist has been completed for this study. This table provides detailed information about our intervention^21^.

### Outcomes and variable assessment

Primary outcome measures included: participants' healthy lifestyle behaviors; daily energy intake; weight and body mass index.

Secondary outcome measures included participants´ adherence to the project; neck, arm, waist and buttock circumferences; fat mass percentage; blood pressure; fasting blood glucose and serum lipid profile.

The assessment of primary and secondary outcome measures was performed before randomization (at baseline) and after the intervention (at the end of the 4^th^ month).

The Persian translation of the Health Promoting Lifestyle Profile II (HPLP-II) questionnaire was used to investigate participants' lifestyle behaviors. This questionnaire includes 52 questions in six lifestyle subscales (spiritual growth "11 questions", health responsibility "13 questions", interpersonal relations "8 questions", stress management "6 items", physical activity "7 questions", and nutrition with 7 questions) ^18^. Each question has a four-point response scale to determine the frequency of that behavior, ranging from 1-4; "1” representing "never" and “4” representing "routinely". An average of ≥2.50 was considered to be a positive response in each question. HPLP-II instrument has been translated into different languages ^22, 23^; validity and reliability of its Iranian version have been confirmed ^24, 25^. The alpha reliability coefficient of the Persian translation of this questionnaire was reported 0.82 for the total scale and ranged from 0.64 to 0.91 for its subscales^25^.

Participants´ adherence was defined as participation until the end of study ^26, 27^.

Other data collected included age, gender, education level, occupation, marital status, living region (urban or rural) and history of comorbid physical or mental disorders. Moreover, weight, body mass index (BMI), neck, arm, waist and buttock circumferences; fat mass percentage; blood pressure; fasting blood glucose and serum lipid profile were examined. BMI was calculated as weight (in kilogram)/ height^2^ (in meter) and was classified according to WHO recommended cut-off-points; 18.5-24.9 Kg/m^2^ as normal, 25.0-29.9 Kg/m^2^ overweight and ≥30.0 Kg/m^2^ obese^28^.

Neck circumference was measured around the neck between cervical vertebrae and the thyroid gland in the front of neck; arm circumference was measured around the arm, midpoint between the acromion and the elbow; waist circumference was measured around the midpoint between the lower margin of the last palpable rib and the top of the iliac crest; and buttock measurement was taken at the maximum circumference over the buttocks. All measurements were taken once over light clothing and values were recorded in centimeters.

Fat mass percentage was measured by using hand-to-foot bioelectrical impedance analysis technique with a digital body fat calculator (Omron Company: BF511 Model). This technique has been reported as a simple, quick and non-invasive method which can give reliable measurements of body composition with minimal intra- and inter-observer variability; the results are available immediately and reproducible with <1% error on repeated measurements^29^.

Blood pressure was measured with the participant in sitting position, using a digital sphygmomanometer (Omron M-6 brand). Early morning venous blood samples (5 mL) were collected after fasting for at least 12 h to assess fasting blood sugar (FBS), total cholesterol, high-density lipoprotein (HDL) cholesterol, low-density lipoprotein (LDL) cholesterol, total cholesterol and triglycerides (TG) levels; these values were measured using Pars Azmun kits via autoanalyzer respons®910 DiaSys system. All of laboratory tests have been conducted in a particular laboratory undertaken external quality control.

According to National Cholesterol Education Program Adult Treatment Panel III Report, cut-off points for fasting blood glucose, total serum cholesterol, triglyceride, HDL and LDL levels have been considered less than 126, 200, 150, 40 and 100 mg/dL, respectively^30^.

All of participants were interviewed in two different days using structured 24-h food-recall questionnaires; for each day, a separate questionnaire was completed. This questionnaire is a valid tool to assess dietary intake of study population and has been used in different studies in Iran and other countries ^31-34^. The participants recorded detailed diary of foods and beverages consumed in previous day from early morning until midnight in the given questionnaires. They were explained to record their food diary as defined reference of food measures^32^. In this way, two experienced dieticians interviewed and gathered information about food recipes, preparation method, ingredients and quantity of food intake. Dietary records were analyzed using Nutritionist IV software (modified for Iranian food) and transcribed in terms of total daily energy (kilocalorie), carbohydrate (gram), fat (gram) and protein (gram) intake; in addition to, the proportion of daily energy from carbohydrate, fat and protein were described in the results.

### Sample size

Sample size was estimated^35^ by considering confidence level of 95%, study power of 80%, assumption of S_1_ as 2.5 kg weight loss in high-intensive intervention group and 2 kg weight change in control group (education group), for discovering 1 kg difference in weight change among the three groups; in addition, taking a possible 20% loss to follow-up^27^ into account, 100 individuals were calculated for each group.

### Randomization

To generate the random allocation sequence in these three groups, we allocated study population as sequentially numbered method until each of the three study groups had almost the same number of men and women in the age-groups of 40-49 and 50-60 yr.

A medical doctor (MD, MPH) generated the random allocation sequence, enrolled participants, and assigned participants to interventions.

### Trial registration

This research was registered in the website of Iranian Registry of Clinical Trials (www.irct.ir) as IRCT2015070423055N1 (19769) identification number.

### Statistical methods

Data analysis was performed by SPSS 17 (Chicago, IL, USA); ANOVA, ANOVA repeated measures, chi-square, Fisher's exact test and t-test were used for data analysis with significance level of *P*<0.05. The Kolmogorov-Smirnov test was used to evaluate the normal distribution of quantitative data.

We used paired t-test, ANOVA and ANOVA repeated measures to compare groups for primary and secondary outcomes; in addition to calculating effect size.

Ethics approval and consent to participate

All of participants provided their written informed consent. They have been assured that their information would be kept confidential. This research has been approved in Ethics Committee of Babol University of Medical Sciences with registration code Mubabol.Rec.1394.45.

## Results

Totally, 301 individuals were enrolled in the study: 146 (48.5%) male and 155 (51.5%) female; 145 (48.2%) in the age group of 40-49 yr and 156 (51.8%) in 50-60 yr old group; 177 (58.8%) had education level less than diploma, only 37 (12.3%) had academic education; 286 (95.0%) were married; and 292 (97.0%) were living in urban area of Amirkola. Most of them reported no previous history of physical (235; 78.1%) or mental (288; 95.7%) disorders.

Forty-five people who referred to research center have been excluded because 15 persons were illiterate, 14 cases were prescribed hypoglycemic and/or lipid-lowering agents, 11 persons reported the previous physician-diagnosis of diabetes mellitus, 2 cases did not accept to participate in the study because they should refer to the research center several times during the study, one person did not live in Amirkola, one woman was in breastfeeding period and one man had heart failure and had to limit his physical activities.

Baseline data for primary outcome measures of the three research groups is presented in Table 1. High-intensive intervention group consisted of 55 (51.9%) female and 51 (48.1%) male; low-intensive group 55 (56.1%) female and 43 (43.9%) male; and the control group consisted of 45 (46.4%) female and 52 (53.6%) male (*P*=0.395). Lifestyle behaviors, dietary intake, anthropometric, serum glucose and lipid profile of the participants before and after the intervention are presented in Tables 2 to 4.

**Table 1:** Baseline characteristics of the three research groups

**Variable**	**High-intensive Intervention** ** Group, n=106**		**Low- intensive intervention** **group, n=98**		**Control group** **n=97**		***P***
	**Mean**	**SD**	**Mean**	**SD**	**Mean**	**SD**	
Age (yr)	**49.8**	**5.2**	**49.8**	**5.4**	**49.5**	**5.7**	**0.888**
Weight (kg)	78.2	11.7	77.8	13.7	78.9	13.2	0.813
Body Mass Index (kg/m2)	28.3	4.3	28.2	4.6	28.2	4.3	0.969
Total score of healthy lifestyle behaviors	138.0	17.8	139.3	18.8	136.9	21.0	0.675
Total daily energy intake (kcal)	2135.6	635.5	2171.9	735.5	2237.1	797.5	0.612

**Table 2:** Healthy lifestyle behaviors score in 40-60 year old adults, before and after the intervention, in three research groups

**Lifestyle** **subdomains**	**Before intervention scores**								**After intervention scores**						***P*** **value** ^b^
	**High-intensive** **group**		**Low-intensive** **group**		**Control** **group**		***P*** ** value** ^a^	**High-intensive** **group**		**Low-intensive** **group**		**Control** **group**		***P*** **value** ^a^	
	**Mean**	**SD**	**Mean**	**SD**	**Mean**	**SD**		**Mean**	**SD**	**Mean**	**SD**	**Mean**	**SD**		
Spiritual growth	34.4	4.9	33.6	5.1	33.4	5.3	0.369	37.7	4.7	37.0	4.7	36.7	4.5	0.350	0.001
Health responsibility	33.3	6.9	33.8	7.1	33.0	8.2	0.774	39.9	8.1	38.9	6.7	38.3	6.9	0.380	0.001
Interpersonal relations	23.7	3.9	23.9	4.3	23.8	4.6	0.960	25.5	3.8	25.5	4.2	25.7	4.2	0.948	0.001
Stress management	13.5	3.0	13.9	2.8	13.6	3.0	0.603	16.5	3.1	16.2	3.6	16.2	3.5	0.778	0.001
Physical activity	13.0	4.5	13.4	4.1	12.6	4.8	0.442	17.1	4.9	15.8	5.1	14.9	4.9	0.015	0.001
Nutrition	20.1	3.2	20.6	3.3	20.5	3.1	0.415	24.6	2.8	24.4	2.8	23.6	3.0	0.096	0.001
Total score	138.0	17.8	139.3	18.8	136.9	21.0	0.675	161.3	18.9	157.9	18.7	155.5	18.9	0.138	0.001

^a^ Between groups ^b^ ANOVA repeated measures

**Table 3:** Anthropometric, blood pressure, fasting blood glucose and serum lipid profile in 40-60 yr old adults, before and after the intervention, in three research groups

**Variables**	**Before intervention**								**After intervention**						***P*** **value** ^b^
	**High-intensive intervention group**		**Low-intensive intervention group**		**Control group**		***P*** ** value** ^a^	**High-intensive intervention group**		**Low-intensive intervention group**		**Control** **group**		***P*** ** value** ^a^	
	**Mean**	**SD**	**Mean**	**SD**	**Mean**	**SD**		**Mean**	**SD**	**Mean**	**SD**	**Mean**	**SD**		
Weight (kg)	78.2	11.7	77.8	13.7	78.9	13.2	0.813	77.0	11.6	76.5	13.1	78.2	11.1	0.647	0.001
Body Mass Index (Kg/m^2^)	28.3	4.3	28.2	4.6	28.2	4.3	0.969	28.0	4.2	27.8	4.6	28.2	4.1	0.828	0.001
Neck circumference (cm)	38.2	3.8	37.8	3.7	37.9	4.2	0.721	35.9	3.5	35.5	3.4	36.4	3.4	0.179	0.001
Arm circumference (cm)	32.6	3.2	32.7	3.1	32.6	3.2	0.966	30.9	2.9	30.7	2.8	31.3	2.7	0.378	0.001
Waist circumference (cm)	94.9	9.0	94.3	9.7	93.8	11.4	0.705	89.3	8.5	88.6	10.2	90.1	8.9	0.579	0.001
Buttock circumference (cm)	107.3	10.7	107.1	10.2	106.8	10.2	0.947	103.0	8.8	102.3	7.9	103.0	9.3	0.832	0.001
Fat mass (%)	31.7	10.2	31.8	10.3	31.2	10.6	0.912	32.7	14.8	31.5	10.9	31.5	10.6	0.746	0.001
Systolic blood pressure (mm/Hg)	125.4	18.3	124.7	14.9	124.4	16.5	0.913	118.1	16.1	119.2	15.2	124.1	19.5	0.051	0.092
Diastolic blood pressure (mm/Hg)	80.3	10.9	80.7	8.6	80.7	11.1	0.960	76.9	8.9	77.3	8.7	79.4	9.9	0.193	0.001
Fasting blood glucose (mg/dL)	94.8	21.4	96.1	24.6	104.6	42.4	0.055	98.6	21.8	97.5	25.4	101.3	26.9	0.601	0.001
Serum TG (mg/dL)	171.9	96.2	178.2	140.7	169.5	116.1	0.873	142.7	77.6	134.7	70.1	152.9	88.7	0.338	0.001
Serum total cholesterol (mg/dL)	219.8	50.1	214.0	46.7	217.7	45.4	0.687	203.3	40.8	201.5	40.4	203.4	39.5	0.941	0.001
Serum HDL (mg/dL)	58.0	14.7	56.6	15.2	56.0	14.1	0.617	64.4	14.9	62.3	13.8	62.7	14.9	0.591	0.001
Serum LDL (mg/dL)	120.1	26.3	116.9	27.7	119.3	25.9	0.692	118.9	28.6	119.0	27.0	121.7	27.5	0.773	0.001

^a^ Between groups ^b^ Anova repeated measures

**Table 4:** Dietary intake in 40-60 yr old adults, before and after the intervention, in three research groups

**Variables**	**Before intervention**								**After intervention**						***P*** **value** ^b^
	**High-intensive** **intervention** **group**		**Low- intensive** **Intervention** **group**		**Control** **group**		***P*** ** value** ^a^	**High-intensive** **intervention** **group**		**Low- intensive** **intervention** **group**		**Control** **group**		***P*** **value** ^a^	
	**Mean**	**SD**	**Mean**	**SD**	**Mean**	**SD**		**Mean**	**SD**	**Mean**	**SD**	**Mean**	**SD**		
Total daily energy (kCal)	2135.6	635.5	2171.9	735.5	2237.1	797.9	0.612	1838.9	672.6	1823.4	574.0	1782.8	666.4	0.843	0.001
Daily intake of carbohydrate (gr)	330.4	99.5	348.28	122.8	350.7	125.7	0.401	282.7	118.4	292.2	94.9	281.8	116.5	0.781	0.001
Daily intake of protein(gr)	81.8	30.9	80.1	32.8	84.5	39.3	0.677	75.3	27.2	71.0	28.3	69.3	33.4	0.381	0.001
Daily intake of fat (gr)	54.1	25.8	50.9	25.3	55.1	30.6	0.545	45.2	21.6	41.2	18.4	42.1	19.9	0.350	0.001
Proportion of energy from carbohydrate (%)	62.4	8.4	64.3	7.3	63.2	9.1	0.278	61.0	8.9	64.4	7.1	63.2	8.5	0.016	0.007
Proportion of energy from protein (%)	15.2	3.5	14.8	2.9	15.1	3.6	0.664	16.9	4.3	15.4	3.1	15.5	4.0	0.010	0.124
Proportion of energy from fat (%)	22.4	6.8	20.9	6.7	21.7	7.5	0.347	22.0	6.9	20.1	5.8	21.3	6.3	0.125	0.022

^a^ Between groups ^b^ Anova repeated measures

Mean baseline BMI was in the range of overweight in both sexes (29.2±4.7 in women and 27.3±3.8 kg/m^2^ in men); after intervention, although body mass index had a statistically significant reduction (*P*<0.001), it was still in the overweight range in two sexes (28.9 ±4.7 in women and 26.9 ±3.6 kg/m^2^ in men).

Before intervention, mean of healthy lifestyle behaviors score in the subdomains of spiritual growth, health responsibility, interpersonal relations and nutrition were higher than average; but in the subdomains of stress management and physical activity were in poor condition. After intervention, the mean scores of all the six subdomains of HPLP-II questionnaire had significant improvement (*P*<0.001), furthermore, it has increased to a higher level than the average in the subdomain of stress management. The three research groups had no significant difference in this change in healthy lifestyle behaviors, except physical activity which had significant statistical increase in high-intensive intervention group (*P*=0.015). Furthermore, nutrition score increased after the intervention and this improvement was more in high-intensive intervention group compared to the other two groups; however, this change was not statistically significant (*P*=0.096) (Table 2).

Weight, BMI, neck, arm, waist and buttock size, diastolic blood pressure, serum TG, total cholesterol and HDL levels have been changed to a better condition in comparison with the baseline values (*P*<0.001). Mean of systolic blood pressure had a clinical decline in comparison with the initial condition (decreased from 124.8 ±16.6 to 120.2 ±16.9 mmHg), but this difference was not statistically significant (*P*=0.092). Fat mass, fasting blood glucose and serum LDL levels did not decrease after intervention. Totally, the range of weight change was -5.4-13.8 kg in women and -5.9-15.5 kg in men. This weight change was significantly different between the three groups (*P*=0.020) and high-intensive intervention group had higher weight loss in comparison with the other groups; mean of weight loss was 1.5 ±2.9 (95% CI: 0.90, 2.05) kg in high-intensive group, 1.0 ±2.9 (95% CI: 0.41, 1.63) kg in low-intensive group and 0.3±2.4 (95%CI: -0.26, 0.82) kg in control group. Weight change had no significant difference between the two sexes (*P*=0.981) and age-groups (*P*=0.871) (Table 3).

Table 4 shows that total daily energy; daily intake of carbohydrate, protein and fat decreased, in addition to, the proportion of energy from carbohydrate and fat per day reached to lower values and energy from protein increased after intervention (increased from 15.0% to 15.9% of total daily energy). These changes were statistically significant in total daily energy (*P*<0.001), daily amount of carbohydrates (*P*<0.001), proteins (*P*<0.001) and fat (*P*<0.001), the percentage of energy from carbohydrates (*P*=0.007) and fat (*P*=0.022) but was not significant about the percentage of energy from proteins (*P*=0.124). Three research groups had significant statistical differences in the percentage of daily energy from carbohydrates (*P*=0.016) and proteins (*P*=0.010) after intervention. This change was mainly observed in high- intensive intervention group.

Mean difference of primary outcome measures before and after intervention in three research groups has been represented in Table 5.

**Table 5:** Mean difference of primary outcome measures before and after intervention in three research groups

**Variables**	**Research Group**						***P*** ** value**
	**High-intensive** **intervention group**		**Low-intensive** **intervention group**		**Control** **group**		
	**Mean**	**SD**	**Mean**	**SD**	**Mean**	**SD**	
Difference in healthy lifestyle behaviors score	22.5	20.4	18.2	19.1	17.7	21.0	0.221
Weight difference	1.48	2.86	1.02	2.96	0.28	2.36	0.020
BMI difference	0.53	1.05	0.36	1.03	0.11	0.86	0.021
Difference in total daily energy intake	247.9	598.9	335.2	713.3	439.2	740.7	0.199

## Discussion

Our lifestyle modification program could have positive impact on healthy lifestyle behaviors, dietary intake and weigh in addition to BMI, neck, arm, waist and buttock size, blood pressure and serum lipid profile (serum TG, total cholesterol and HDL values) of middle-aged (40-60 yr old) adults. High intensive intervention group who received weekly group nutrition and physical activity training classes, individual nutrition consultation and educational package in addition to weekly aerobic exercises had better outcome variables especially in weight loss, improving physical activity, decline in the proportion of energy from carbohydrates and improvement in the proportion of energy from proteins.

Our result in outcome measures has similarities with some other studies. Structured lifestyle interventions could be effective in reduction of diabetes incidence and cardiovascular risk^13^. Lifestyle modification could reduce the incidence of diabetes mellitus more than the standard treatment plans in prediabetic adults; furthermore, this lifestyle adaptation could provide better glycemic control, improvement in the capacity for physical activity and increase in the weight reduction^12^. The effectiveness of lifestyle interventions was reported in the weight loss (mean of 8.9 kg, 95% CI: 7.7-10.2) and 2.8 Kg/m^2^ reduction in BMI level in the persons with high grades of obesity; and suggested that longer duration of interventions could induce more weight loss. A significant reduction was showed in fat mass, waist, blood pressure, total cholesterol, LDL and triglycerides, but did not have significant impact on HDL and fasting blood glucose levels. Lifestyle interventions which had physical activity component could improve the weight and cardiometabolic risk factors in obese persons^14^. Lifestyle interventions had efficacy to reduce cardiovascular risk factors, at least as drugs. He mentioned that most lifestyle interventions in developing countries enrolled a specific group of participants, rather than whole community^37^. Trying to avoid that pattern, we used a sample population selected from healthy middle-aged (apparently non-patient 40-60 yr) adults.

In our study, weight changes before and after the intervention in the three target groups, two age-groups and two sexes showed that after intervention, mean weight reduction was higher in high-intensive intervention group, and the two intervention groups had higher weight loss in comparison with the control group. High-intensive group had better outcomes (especially in physical activity, reduction in the percentage of energy from carbohydrates and increase in the percentage of energy from proteins) than the other groups. Mean weight loss was 1.5, 1.0 and 0.3 kg, in high-intensive, low-intensive and control groups respectively. These values ​​have similarities and differences with previous studies, which may be due to characteristics of the sample, duration of intervention, habitual and cultural differences and the content of interventions. Lifestyle interventions, with a duration of at least 12 wk, in severe obese adults could induce more weight reduction in comparison with control groups who received basic care or standard care. The range of weight loss in his study was reported 1.0-11.5 kg; in addition, lifestyle interventions with combined nutrition and physical activity components would provide higher level of weight reduction^38^. The weight loss intervention programs was reviewed with duration ≥12 wk, reported a range of 16 wk to 9 yr of time duration in these interventions; mean age of participants in his study was 55 (47-60) yr, and most of interventions were held as face-to-face group classes or individual consultation sessions. Mean weight reduction in his study was reported 3.33 (95% CI: -5.06-1.60) kg^39^. Obese adults could have had 6-8 Kg (nearly 6-8% of the baseline) weight loss if they had participated in high-intensive lifestyle interventions which had ≥14 treatment visits and had undertaken diet regulation, physical activity promotion, and behavior therapy^7^. High-intensive interventions which took time more than six months, and included low-calorie diet, high physical activity and behavior therapy could provide 8 kg weight reduction (8% of initial weight); furthermore, their quality of life would be improved^15^. Published manuscripts were reviewed in the yr 1989-2013 about the effectiveness of lifestyle educational interventions on biochemical variables in high-risk cardiovascular adults, concluded that among 43 reviewed articles, only 2 manuscripts reported no significant change in the risk factors after intervention, and the other articles demonstrated a significant improvement in cardiovascular risk factor following lifestyle educational interventions^40^.

In our study, although a significant weight reduction was observed at the end of study in two intervention groups compared to the control group, since the last month of intervention (from 21/3/2017 to 20/4/2017) coincided with Nowruz (the first month of the new year), this subject might have had an impact on our outcome measures (made lower weight reduction); because in the first 13 d of each new year in Iran, people are often on holiday and their nutrition and physical activity programs are usually different with other months. Another factor which we found during the implementation of intervention classes was unauthorized activity of traditional educators in this region which triggered some people to have unhealthy nutrition programs (such as intake of animal fat instead of vegetable oils, untreated salt consumption and low intake of dairy products and vegetables).

We targeted non-patient middle-aged adults for this research. This age group is a productive group, biologically and economically. If they become ill, the most work days will be lost. Other age-groups in family and society are dependent on this age-group. Their mortality, morbidity and behaviors have an impact on all healthy aspects of the family. Their life-threatening risk factors, such as obesity, diabetes mellitus, hypertension and other cardiometabolic disorders could have impact on the health status of other family members. Chronic disorders in this age-group can inhibit healthy active ageing. The other strength points of our study were population-based conduction of the project; selection and enrollment of the same from two sexes and two age-groups (40-49 and 50-60 yr) which provided better grounds for comparison.

Despite the need for frequent referrals of the participants for before-after examinations and participation in several intervention sessions, 266 individuals (88.4%) had adherence to the program. This value is higher than the adherence rate reported in Tehran, Iran in which 20% of participants in intervention group and 33.7% of control group did not complete the program. In our study, loss to follow-up rate was respectively, 5% and 8% in two intervention groups and 22% in control group. In Netherland, which assessed the adherence rate of >50 yr old adults in health-promoting interventions (web-based and paper-based contents), paper-based interventions induced more adherence rate (19% in comparison with 12% of web-based ones)^27^. Better adherence in our study may be attributed to our study design; the proper location of research center and rented sport hall – in the center of study region- which facilitated easy access of the participants to have continuous and regular referring for different steps of study program. Moreover, having appropriate communication with people, presence of a medical doctor in all stages of training sessions and individual consults, calling the participants by phone to recall the time of visits or sessions and assigning a mobile phone line for people calls can justify this result.

Limitations of this study were the duration needed to complete total sample size (especially to inform and engage the male population of the region). Furthermore, religious and cultural occasions (such as Ramadan, Muharram and Nowruz) in which lifestyle behaviors (diet and physical activity) change were located at time period of our study. During the month of Ramadan, Muslims have a meal before sunrise and fast until the sunset; and in the month of Muharram, especially in the first decade of Muharram, Muslims usually attend in mosques and other religious places and eat special foods such as oily rice with meat, and this change might have had an impact on the outcome measures.

In addition to, we focused our intervention to two (nutrition and physical activity) out of six domains of health-promoting lifestyle behaviors. It is suggested to other researchers to design proper interventional studies for all of these subdomains.

## Conclusion

Our intervention program had a positive impact on healthy lifestyle behaviors, dietary intake and weigh in addition to some other anthropometric variables, blood pressure and serum lipid profile of middle-aged (40-60 yr) adults.

## Acknowledgements

This study has been approved in Babol University of Medical Sciences with the registration code 2786. Hereby, the financial support of the Vice-Chancellor for Research and Technology of Babol University of Medical Sciences, and the cooperation of the people who participated in the study are greatly appreciated.

## Conflict of interest statement

The authors declare that there is no conflict of interests.

## Funding

This study has been supported financially by the Vice-Chancellor for Research and Technology of Babol University of Medical Sciences.

## Highlights

- All of the six subdomains of health-promoting lifestyle profile have been improved.
- This study could have positive impact on some important anthropometric measures.
- The range of weight change was -5.4-13.8 in women and -5.915.5 kg in men.
- Serum triglyceride, total cholesterol, and HDL levels changed to a better condition.
- Total daily energy had positive changes after the intervention
